# Response of an Artificial Filled Fracture with Varied Water Contents to Dynamic Stressing

**DOI:** 10.3390/s26195992

**Published:** 2026-09-22

**Authors:** Ning Liang, Xiaolin Huang, Shengwen Qi, Yifang Huang

**Affiliations:** 1 State Key Laboratory of Lithospheric and Environmental Coevolution, Institute of Geology and Geophysics, Chinese Academy of Sciences, Beijing 100029, China; liangning@mail.iggcas.ac.cn; 2 Department of Civil Engineering, School of Human Settlements and Civil Engineering, Xi’an Jiaotong University, Xi’an 710049, China; 3 School of Software Engineering, Faculty of Electronic and Information Engineering, Xi’an Jiaotong University, Xi’an 710049, China; huang_yifang@yeah.net

**Keywords:** filled fracture, dynamic stressing, trapped-unloading response, viscosity, dynamic weakening of gouge-filled faults

## Abstract

Field observations indicate that gouge-filled faults can be weakened under dynamic stressing and further trigger earthquakes. Previous studies have qualitatively attributed such fault weakening to elevated pore pressure within fault gouges induced by seismic deformation, which reduces fault shear strength and frictional resistance. However, direct experimental evidence and systematic quantitative analyses clarifying the hydromechanical controls on the dynamic weakening process remain scarce. Here, artificial filled fractures were prepared by sandwiching granular infills with varied water content between two cylindrical aluminum bars to constitute an aluminum split Hopkinson pressure bar (SHPB) system. Benefiting from the rock-like wave impedance of aluminum bars, this setup can better reproduce real in situ dynamic loading conditions. Using this system, we investigated the hydromechanical response of filled fractures under dynamic stressing and clarified the underlying fault weakening mechanism. The experimental results indicate that the filled fractures undergo significant compression under dynamic loading and exhibit limited rebound deformation during unloading, presenting a distinctive trapped-unloading behavior and obvious stiffness enhancement relative to the loading stage. Variations in the water content of fracture infills substantially alter the stiffness, viscosity, and stress relaxation properties of the filled fractures, thereby regulating stress wave propagation and dynamic deformation characteristics. It is inferred that higher water content significantly hinders the dissipation of excess pore water pressure, reduces the effective confining stress, and ultimately intensifies the hydromechanical weakening of filled fractures. These quantitative findings provide novel experimental insights into the hydromechanical weakening mechanism of natural gouge-filled faults under dynamic stressing.

## 1. Introduction

A fracture is any separation in a geologic formation and can occur at all scales. Generally, there are two main types of fractures in a natural rock mass: joints and faults [1,2,3]. When a stress wave impinges on a fracture, on the one hand, reflection, transmission and wave-type conversion take place [2,4,5]. On the other hand, the fracture produces a dynamic deformation response, such as compression, opening and slip, which influences the stability of the engineering rock mass and even the crust [6,7,8]. It is thus of great significance to explore the response of filled fractures to dynamic stressing for seismology, earthquake engineering and rock engineering.

A joint is a geological discontinuity that has little or no transverse displacement along the fracture surface and can usually be traced for tens to hundreds of meters [1,2,3]. Due to weathering effects, a natural joint is often filled with granular materials such as quartz sand, silt, breccia, gouge and mylonite [9,10]. The infill can have a thickness ranging from microns to several centimeters, which noticeably influences the mechanical behavior of the filled fracture [9,11,12]. Moreover, the fracture is the migration path for groundwater. The water in the fracture gap can prominently affect the deformation and strength characteristics of the filled fracture [13]. Li and Ma [14] investigated the dynamic response of artificial filled fractures with varied water contents using a rock split Hopkinson pressure bar (SHPB) system. They found that the filled fracture produced nonlinear deformation in the loading stage and exhibited hysteresis deformation in the unloading stage. Their results showed that the dynamic response of the filled fracture significantly depended on the water content of the infill. Based on their experimental data, one group of researchers used nonlinear elastic models (hardening in compression) to reproduce the stress wave transmission through a filled fracture [15,16,17]. However, other researchers have claimed that due to the fluid coupling effect, the humid filled fracture produced a viscoelastic response to the stress wave, which should be characterized by viscoelastic models [5,18,19,20,21,22,23]. Zhu et al. [24] found that both the Kelvin viscoelastic model (KVM) and the Maxwell viscoelastic model (MVM) could reproduce stress wave transmission through filled fractures in the frequency domain well. The identical experimental transmitted wave was also reproduced by particle flow numerical simulation considering the KVM of the contact [25]. Based on the above, it is confusing that the dynamic response of the filled fracture can be characterized by both the KVM and MVM since these two models represent very different deformation behaviors. Compared with the nonlinear deformation effect, there are very limited lines of experimental evidence to validate the viscoelastic dynamic response of filled fractures with varied water contents.

A fault is often far larger than a joint, which can usually extend for tens of kilometers [1,2,3]. Distinguished from the joint, a fault experiences considerable shear displacement along the fracture surface. Due to the grinding and crushing as well as weathering effects, the adjacent host rock turns into granular gouge with small particle sizes. In general, the infill has a thickness of more than several meters. A gouge-filled fault often bears tectonic stress and stores a great amount of strain energy. Once it loses stability, the stored strain energy is suddenly released, which can trigger earthquakes. Several lines of evidence have shown that gouge-filled faults weaken due to dynamic stressing associated with the passage of seismic waves and trigger earthquakes [26,27,28,29,30,31,32,33]. Dynamic weakening of gouge faults has been attributed to several factors, including thermal pressurization, hydrodynamic effects, extreme frictional weakening at high slip velocities, acoustic fluidization, and changes in damage zone permeability [34,35,36,37,38,39,40,41,42,43,44,45,46,47]. Nevertheless, except for the hydrodynamic effect, specialized conditions are required to induce fault weakening, i.e., large and fast dynamic loads for thermal pressurization and extreme frictional weakening and resonance frequency for acoustic fluidization. In terms of the hydrodynamic effect, it has been qualitatively concluded that elevated pore pressure of granular gouges by seismic deformation response reduces shear strength and further weakens gouge-filled faults [26,38,40]. Hydrodynamic fluidization under oscillatory viscous flows would be a possible driving mechanism for dynamic weakening of granular materials [48]. Shelly et al. [33] found that a fault produced creep deformation after the passage of a seismic wave and delayed the dynamic triggering of tremors and earthquakes. However, thus far, the viscoelastic response of gouge-filled faults to dynamic stressing has rarely been documented to clarify the hydrodynamic effects on fault weakening.

Although filled joints and the gouge-filled faults are different in scale, they can both be modeled as a sandwich structure, as shown in Figure 1. It is composed of two stiff rock blocks and a layer of soft granular materials mixed with water. It is believed that filled joints and gouge-filled faults should have similar physics in response to stress waves. As mentioned, three controversial issues need to be settled: (1) Whether filled fractures exhibit viscoelastic responses to stress waves. (2) If this response exists, what viscoelastic models are applicable to characterize its dynamic response? (3) Whether a gouge-filled fault produces a viscoelastic response to dynamic stressing and how this process further favors hydrodynamic weakening. The objective of this paper is to explore the response of filled fractures to dynamic stressing and further provide new implications for the hydrodynamic weakening of actual gouge-filled faults.

This paper is structured as follows: Section 1 reviews the previous studies on the dynamic response of filled fractures with different scales and analyzes the limitations of these studies. Section 2 describes the preparation of the artificial filled fracture, the dynamic stressing setup, and analytical models. Section 3 shows the experimental and predicted results by different models, and these results are discussed in Section 4. Conclusions are given in Section 5.

## 2. Materials and Methods

### 2.1. The Artificial Filled Fracture and Dynamic Stressing Setup

Fine granular material (quartz sand) was used to simulate the infill. The particle density is 2984 kg/m^3^, and the grain size distribution ranges from 0.015 to 0.3 mm, with a median effective diameter of 0.15 mm (Figure 2). The water content is reported as gravimetric water content in the current study. The dry bulk density is 1750 kg/m^3^. At a gravimetric water content of 23.63%, the void ratio is 0.705, and the degree of saturation after compaction is 100%. Samples with varied water contents were considered, i.e., 0% (dry), 4.73%, 9.45%, 14.18%, 18.90%, and 23.63% (saturated). Table 1 shows the physical properties of the infill related to the current study. We applied aluminum alloy to simulate the host rock since it has a high-quality factor and similar impedance to natural rock. As shown in Figure 3a, granular materials were placed between two short cylindrical bars made of 7075 aluminum alloy to simulate the sandwich structure of the natural gouge-filled fault or the filled fracture. These two short bars both have a diameter of 50 mm and a height of 50 mm. To prevent the outflow of the granular materials, an aluminum alloy tube holder with an inner diameter of 50.1 mm and an external diameter of 70.1 mm was applied as a sleeve to the sandwich structure. Granular materials were deformed into thin layers to simulate the natural setting of fault gouges. Each sample was pre-compacted 8 times by a standard compactor dropping from the same height to ensure a similar dense state. The average thickness of the infill was 2.5 mm.

The sample assembly was loaded dynamically by an aluminum alloy split Hopkinson pressure bar (SHPB) system (Developed by Institute of Geology and Geophysics, Chinese Academy of Sciences, Chaoyang District, Beijing, China), as shown in Figure 3b. The dynamic stressing setup is mainly composed of a gas gun, a striker bar, an input bar and an output bar. These three bars have the same diameter of 50 mm but different lengths of 2500 mm for the input bar, 2500 mm for the output bar and 400 mm for the striker bar. All bars are made of 7075 aluminum alloy with a density of 2810 kg/m^3^, Young’s modulus of 71.7 GPa and longitudinal velocity of 5050 m/s. Vacuum grease was uniformly applied on contact surfaces to reduce undesired friction. Pressurized air was initially stored in the gas gun to accelerate the striker bar. Due to the impact between the striker bar and the input bar, a stress pulse was generated to dynamically load the artificial filled fracture. The amplitude of the generated stress pulse depended on the air pressure in the gas gun. For better comparison, the initial pressure in the gas gun was fixed at 0.16 MPa to induce similar incident waves.

Note that the stress state in the sample is essentially one-dimensional strain due to the large difference in the modulus between the sample and the holder [44]. Based on the one-dimensional stress theory, we can determine the histories of the strain *ε*(*t*) and stress *σ*(*t*) within the sample [49].

(1) $$\varepsilon \left(t\right)=\frac{C}{L}\int_{0}^{t}\left(\varepsilon_{inc}-\varepsilon_{ref}-\varepsilon_{tra}\right)dt$$

(2) $$\sigma \left(t\right)=\frac{1}{2}E\left(\varepsilon_{inc}+\varepsilon_{ref}+\varepsilon_{tra}\right)$$

where *ε* denotes the strain; *σ* is the stress; *C* is the longitudinal wave velocity of the aluminum bar; *L* is the thickness of the sample; *E* is the Young’s modulus of the aluminum bar; and the subscripts “*inc*”, “*ref*” and “*tra*” denote the incident, reflected and transmitted waves, respectively.

### 2.2. Presumed Constitutive Equations of the Filled Fracture by Dynamic Stressing

According to the experimental data, we can acquire dynamic stress histories before and after loading the filled fracture as well as their dynamic stress–strain relations. However, it is often insufficient to clarify the response of a filled fracture to dynamic stressing from the viewpoint of the physical mechanism. We thus presumed some potential constitutive equations to forward-fit the experimental data and then made comparative evaluations to clarify the dynamic constitutive characteristics of the filled fracture.

We consider the *x*-*z* plane, where the filled fracture (planar) coincides with the *x*-axis. Due to the inertia effect of the infill, the stress relations between two contact surfaces of the filled fracture can be characterized by the following two equations [24]:

(3) $${\tilde{\sigma }}_{zx}^{-}-{\tilde{\sigma }}_{zx}^{+}=-\omega^{2}m_{t}{\tilde{u}}_{x}^{+}$$

(4) $${\tilde{\sigma }}_{zz}^{-}-{\tilde{\sigma }}_{zz}^{+}=-\omega^{2}m_{n}{\tilde{u}}_{z}^{+}$$

where the “~” symbol denotes the Fourier transform; the “+” and “−” symbols denote two contact surfaces of the fault; *σ*_zx_ and *σ*_zz_ are the shear and normal stresses; *u*_x_ and *u*_z_ are the displacements in the *x* and *z* directions; *ω* denotes the angular frequency; *m*_n_ = *ρ_f_d* is the normal mass of the infill that determines the stress discontinuity in the normal direction, where *ρ_f_* is the density of the infill and *d* is the filled thickness; and *m*_t_ is the tangential mass of the infill, which determines the stress discontinuity in the tangential direction. For the case of the normally incident longitudinal wave, we can have *m*_t_ = *m*_n_.

When dynamically stressed, the displacements between two fracture contact surfaces are discontinuous, which results in deformation of the filled fracture. Currently, it is not clear to know specific dynamic constitutive relations. Therefore, we presumed three potential models in advance, i.e., the purely elastic model (PEM), KVM, and MVM, since they can basically characterize the typical mechanical response of geological materials such as recoverable deformation, elastic after effect, and stress relaxation.

For the PEM (one spring), the displacement of the fracture is equal to the stress divided by the elastic stiffness.

(5) $${\tilde{u}}_{x}^{-}-{\tilde{u}}_{x}^{+}=\frac{{\tilde{\sigma }}_{zx}^{+}}{k_{s}}$$

(6) $${\tilde{u}}_{z}^{-}-{\tilde{u}}_{z}^{+}=\frac{{\tilde{\sigma }}_{zz}^{+}}{k_{n}}$$

where *k*_n_ and *k*_s_ denote the normal and shear elastic stiffness of the filled fracture, respectively.

Equations (3)–(6) were written as the matrix form

(7) $${\left[\begin{array}{c} \begin{array}{c} {\tilde{u}}_{x}\\ {\tilde{u}}_{z} \end{array}\\ {\tilde{\sigma }}_{zz}\\ {\tilde{\sigma }}_{xz} \end{array}\right]}^{+}={\left[K\right]}_{P}{\left[\begin{array}{c} \begin{array}{c} {\tilde{u}}_{x}\\ {\tilde{u}}_{z} \end{array}\\ {\tilde{\sigma }}_{zz}\\ {\tilde{\sigma }}_{xz} \end{array}\right]}^{-}$$

where ${\left[K\right]}_{P}=\left[\begin{array}{c} \begin{array}{c} 1\\ 0 \end{array}\\ 0\\ \omega^{2}m_{t} \end{array}\begin{array}{c} \begin{array}{c} 0\\ 1 \end{array}\\ \omega^{2}m_{n}\\ 0 \end{array}\begin{array}{c} \begin{array}{c} 0\\ 1/k_{n} \end{array}\\ 1\\ 0 \end{array}\begin{array}{c} \begin{array}{c} 1/k_{s}\\ 0 \end{array}\\ 0\\ 1 \end{array}\right]$.

For the KVM (one spring and one dashpot in parallel), the two elements have the same displacement. The total stress of the model is equal to the sum of the stresses between the two elements. The displacement discontinuity conditions for the KVM can be expressed as

(8) $${\tilde{u}}_{x}^{-}-{\tilde{u}}_{x}^{+}=\frac{{\tilde{\sigma }}_{zx}^{+}}{k_{s}+j\omega \eta_{s}}$$

(9) $${\tilde{u}}_{z}^{-}-{\tilde{u}}_{z}^{+}=\frac{{\tilde{\sigma }}_{zz}^{+}}{k_{n}+j\omega \eta_{n}}$$

where $j=\sqrt{-1}$ is the imaginary unit and *ƞ*_n_ and *ƞ*_s_ stand for the normal and shear viscous coefficients of the filled fracture, respectively.

Equations (3), (4), (8) and (9) were written as

(10) $${\left[\begin{array}{c} \begin{array}{c} {\tilde{u}}_{x}\\ {\tilde{u}}_{z} \end{array}\\ {\tilde{\sigma }}_{zz}\\ {\tilde{\sigma }}_{xz} \end{array}\right]}^{+}={\left[K\right]}_{K}{\left[\begin{array}{c} \begin{array}{c} {\tilde{u}}_{x}\\ {\tilde{u}}_{z} \end{array}\\ {\tilde{\sigma }}_{zz}\\ {\tilde{\sigma }}_{xz} \end{array}\right]}^{-}$$

where ${\left[K\right]}_{K}=\left[\begin{array}{c} \begin{array}{c} 1\\ 0 \end{array}\\ 0\\ \omega^{2}m_{t} \end{array}\begin{array}{c} \begin{array}{c} 0\\ 1 \end{array}\\ \omega^{2}m_{n}\\ 0 \end{array}\begin{array}{c} \begin{array}{c} 0\\ 1/\left(k_{n}+j\omega \eta_{n}\right) \end{array}\\ 1\\ 0 \end{array}\begin{array}{c} \begin{array}{c} 1/\left(k_{s}+j\omega \eta_{s}\right)\\ 0 \end{array}\\ 0\\ 1 \end{array}\right]$.

For the MVM (one spring and one dashpot in series), the two elements have the same stress. The total displacement of the model is equal to the sum of the displacements between two elements. From these relations, we can derive the displacement discontinuity conditions for the MVM as

(11) $${\tilde{u}}_{x}^{-}-{\tilde{u}}_{x}^{+}=\left(\frac{1}{k_{s}}+\frac{1}{j\omega \eta_{s}}\right){\tilde{\sigma }}_{zx}^{+}$$

(12) $${\tilde{u}}_{z}^{-}-{\tilde{u}}_{z}^{+}=\left(\frac{1}{k_{n}}+\frac{1}{j\omega \eta_{n}}\right){\tilde{\sigma }}_{zz}^{+}$$

Equations (3), (4), (11) and (12) were written as

(13) $${\left[\begin{array}{c} \begin{array}{c} {\tilde{u}}_{x}\\ {\tilde{u}}_{z} \end{array}\\ {\tilde{\sigma }}_{zz}\\ {\tilde{\sigma }}_{xz} \end{array}\right]}^{+}={\left[K\right]}_{M}{\left[\begin{array}{c} \begin{array}{c} {\tilde{u}}_{x}\\ {\tilde{u}}_{z} \end{array}\\ {\tilde{\sigma }}_{zz}\\ {\tilde{\sigma }}_{xz} \end{array}\right]}^{-}$$

where ${\left[K\right]}_{M}=\left[\begin{array}{c} \begin{array}{c} 1\\ 0 \end{array}\\ 0\\ \omega^{2}m_{t} \end{array}\begin{array}{c} \begin{array}{c} 0\\ 1 \end{array}\\ \omega^{2}m_{n}\\ 0 \end{array}\begin{array}{c} \begin{array}{c} 0\\ (1/k_{n}+1/(j\omega \eta_{n})) \end{array}\\ 1\\ 0 \end{array}\begin{array}{c} \begin{array}{c} (1/k_{s}+1/(j\omega \eta_{s}))\\ 0 \end{array}\\ 0\\ 1 \end{array}\right]$.

Equations (7), (10) and (13) give the specific boundary conditions of the filled fracture characterized by three presupposed models. On this basis, we can establish corresponding analytical models for the dynamic response of the filled fracture according to the similar method by Huang et al. [50], only needing to substitute the boundary conditions with Equations (7), (10) and (13). The theoretical derivations and detailed elaborations of the relevant methods are provided in Appendix A. Then, the feasibility of the three presupposed models can be systematically evaluated by forward-fitting the experimental data. For the analytical model, the incident wave has to be transferred to the frequency domain. The start and end times to oscillate have 2000 μs of delay and extension, respectively. Thus, the two ends of the signal in the time domain have zero oscillation, which can eliminate the aliasing effect as much as possible in the processing of the fast Fourier transform [50]. Experimental incident waves were fitted by Fourier series expansion (8 orders) and used as the incident waves to predict the reflected and transmitted waveforms in theoretical analysis. By trial and error, the parameters of the three presumed models were fitted until the predicted transmitted wave matched the experimental result to the maximum extent. As the experiment mainly involves one-dimensional deformation, only the normal elastic stiffness and viscous coefficient were involved in the current study.

## 3. Results

### 3.1. Stress Waveforms

For a fixed set of loading and material conditions, three parallel repeated dynamic impact tests were carried out. Figure 4 shows the recorded incident, reflected, and transmitted stress waveforms under the test condition of infill water content of 18.9% and gas driving pressure of 0.16 MPa. It can be observed that both the amplitude and arrival time of the incident, reflected, and transmitted waveforms agree well across the three repeated runs. The relative discrepancies among these repeated trials are within 15%, which demonstrates that the dynamic impact tests for the filled fracture possess acceptable repeatability. Figure 5 shows experimental stress waveforms when the water content of the infill is 0% (dry), 9.45%, and 23.63% (saturated). According to the analytical models in Section 2.2, we obtained forward-fitted results that matched the experimental results to the maximum extent. The fitted properties for the different models are listed in Table 2.

The negative component of the stress waveform is related to the compressive stress, and the positive component denotes the tensile stress. The duration and amplitude of each incident stress wave are approximately 300 μs and 9 MPa, respectively, when the gas gun suddenly releases air at a pressure of 0.16 MPa. As the longitudinal wave velocity of the aluminum bar is 5050 m/s, the wavelength can be calculated as approximately 1500 mm, which is far larger than the thickness of the filled fracture of 2.5 mm. This ensures good stress wave transmission through the filled fracture. Due to the discontinuous deformation of the filled fracture, the stress wave propagation is significantly altered, of which only one part can be transmitted while one part is reflected. Generally, the experimental reflected waveform consists of a larger positive part but a smaller negative part. With increasing infill water content, the stress wave transmission first decreases and then increases. However, the situation is the opposite for the stress wave reflection. For the PEM, the negative part of the predicted reflected wave is much lower than the experimental part, and the predicted transmitted wave has a longer pulse duration. For the KVM, the forward-fitted reflected wave is very different from the experimental result in both amplitude and waveform. That is, there is basically no negative part in the predicted reflected wave, and its duration is slightly broader than the experimental duration, although the transmitted waves are basically consistent. For the MVM, both the predicted reflected and transmitted waves agree well with the experimental waves. Table 3 presents the prediction accuracy of the three constitutive models for reflected and transmitted stress waves, evaluated by R^2^ (coefficient of determination) and NRMSE (normalized root mean square error) calculated from Figure 5. Overall, considering both reflected and transmitted waves, the MVM achieves the best comprehensive prediction performance among the three models, outperforming both the PEM and KVM, as reflected by its generally higher R^2^ and lower NRMSE. The above suggests that stress wave propagation through a gouge-filled fault should be characterized by the MVM, even for the case of dry infill. To further examine this conclusion, the stress–strain relation of the filled fracture should be analyzed.

### 3.2. Stress–Strain Relations and Trapped-Unloading Response

The dynamic stress–strain relations of filled fractures can be obtained according to stress histories and Equations (1) and (2). Figure 6 presents the time–history curves of incident stress, reflected stress, and transmitted stress obtained from the SHPB test on the filled fracture. The superposed curve of incident plus reflected stress (Incident + Reflected) is also plotted for comparison. It can be observed that the temporal evolution and amplitude of the superposed stress (Incident + Reflected) are generally consistent with those of the transmitted stress. This indicates that the stress-equilibrium condition is satisfied at the front and rear interfaces of the filled fracture during dynamic loading. The result verifies that a uniform one-dimensional stress state is achieved, which fulfills the fundamental assumption of one-dimensional stress-wave propagation for the SHPB technique. Accordingly, it provides a solid experimental foundation for calculating the stress–strain response of the filled fracture based on the one-dimensional wave theory and guarantees the validity and rationality of the subsequently derived stress–strain curves. Figure 7 shows the results corresponding to the water content of the infill ranging from 0% (dry) to 23.63% (saturated). In the loading stage, the dynamic normal stress increases with the strain in a nearly linear manner. In the unloading stage, all filled fractures produced obvious hysteresis deformation. The unloading stress–strain curve deviates from the loading curve and drops in a nearly vertical manner. Note that the rebounded strain in the unloading stage is very limited, which suggests that the filled fracture was basically trapped, with dissipation of the dynamic compression stress due to the passage of the stress wave. This dynamic response exhibits an apparent behavior similar to a stress relaxation process.

Figure 7 also shows that the water content of the infill has a prominent effect on the dynamic stress–strain relation of the filled fracture. As the water content increases, the peak stress suffered by the infill first decreases and then increases again, although incident stress waves basically have a similar amplitude. The maximum strain experienced by the infill first increases and then decreases with increasing water content.

Figure 8 shows the experimental and predicted stress–strain relations of the filled fracture as the water content of the infill is 14.18%. For the PEM, the loading stress increases with the strain in a linear manner, which evidently deviates from the experimental result. Compared with the experimental result, the hysteresis phenomenon of the stress–strain curve is not obvious. The unloading stress also varies in a linear manner, and the unloading curve is very close to the loading curve. For the KVM, the predicted dynamic stress–strain curve also obviously deviates from the experimental result. The loading stress increases nonlinearly with strain, and the dynamic stress–strain curve is concave toward the strain axis. When unloading, the strain of the filled fracture still increases, which is distinguished from the experimental result. For the MVM, it clearly shows that the predicted dynamic stress–strain relation has good consistency with the experimental result, especially for the trapped unloading phenomenon. Therefore, the MVM can simulate the trapped-unloading response well.

According to the results in Figure 7, the moduli of the linear part in both the loading and unloading curves were obtained and plotted in Figure 9. The loading modulus in Figure 9a first decreases with increasing infill water content and then increases. The minimum value occurs when the infill has a water content of 9.45%. The unloading modulus in Figure 9b exhibits a generally similar trend, and for most water-content conditions its magnitude is approximately one order of magnitude higher than the loading modulus.

### 3.3. Transmission Reflection Coefficients and Properties Related to Trapped-Unloading Response

The aforementioned results present the dynamic stress–strain response of the filled fracture and the effect of the water content of the infill. In this section, we explore the effect of water content variation on the reflection and transmission of the stress wave and properties related to trapped-unloading response. Figure 10 shows the stress wave transmission and reflection coefficients through the filled fracture versus the water content of the infill. Herein, the reflection coefficient and transmission coefficient are defined as the absolute values of the ratio of the reflected-wave and transmitted-wave amplitudes to the incident-wave amplitude, respectively. The transmission coefficient first decreases and then increases, which is in line with the variation in the modulus in Figure 9. The reflection coefficient first increases with the water content of the infill and then decreases. The results predicted by the MVM are consistent with the experimental results.

Figure 11 shows fitted properties related to stress relaxation by the MVM according to the experimental data. Both the elastic stiffness and viscous coefficient first decrease and then increase as the water content of the infill increases. The water content of the infill corresponding to the minimum magnitude is 9.45%. This variation trend with the water content of the infill is similar to those in Figure 9 and Figure 10a.

## 4. Discussion

As mentioned in Section 3, we provide experimental evidence and a theoretical model of the trapped-unloading response of a filled fracture to dynamic stressing. This is important for understanding the seismic response of natural gouge-filled faults, such as the dynamic loading/unloading deformation response, seismic wave propagation and dynamic fault weakening.

### 4.1. Trapped Unloading Effect Related to Stress Relaxation

As shown in Figure 7 and Figure 8, the nonlinear deformation effect of the filled fracture in the loading stage is less obvious than those in the studies by Li and Ma [14] and Wu et al. [51]. The reason is that the infill in our study was pre-compacted and had a far smaller mean grain size (0.015~0.3 mm) than that in Wu et al. [51] (1~2 mm). For an actual gouge-filled fault, the granular infill is often compacted and has a very small grain size (0.001~0.1 mm) [52]. Hence, it can be inferred that the pre-compaction nonlinear deformation should be inconspicuous. From Figure 7 and Figure 8, it is likely that a filled fracture was prominently stiffened after dynamic stressing since the unloading modulus was one order of magnitude larger than the loading modulus. This resulted from the trapped unloading effect by stress relaxation that can be characterized by the MVM. When loaded by a compressive stress wave, initially, there was not enough time for the dashpot to produce deformation. The deformation was mainly afforded by the spring. Thus, a filled fracture had a compressive stress in a short time and deformed in a nearly linear manner (see Figure 7). In the unloading stage, stress relaxation occurred; that is, deformation of the filled fracture was very limited, while the compressive stress gradually decreased to 0 with the passage of the stress wave. This process was equivalent to the stiffening deformation of the filled fracture compared with that in the loading case. It was found that the dominant frequency of a stress wave decreased when propagating through a fracture [5]. A fracture with a smaller stiffness can reduce the high-frequency component more under the same conditions. Because the MVM can simulate the unloading stiffening deformation effect but the PEM cannot, the frequency decrease in the stress wave by the MVM was less than that of the PEM. We can now understand that the duration of the transmitted wave by the MVM is narrower than that by the PEM and is basically in line with the experimental data. Note that the KVM can also capture transmitted waves that match well with the experimental waves (see Figure 5). Nevertheless, this is only numerical matching rather than the actual physics because the KVM loses the capability of modeling the reflected wave and the stress–strain relation (see Figure 8).

The underlying reason lies in its parallel spring-dashpot configuration. In the KVM, the spring and dashpot are constrained to undergo identical strain, and the total stress is the sum of their elastic and viscous stresses; thus, they must deform synchronously and cannot move independently. When a transient pulse load enters the unloading stage, the applied total stress begins to decrease, and the elastic rebound stored in the spring tends to reduce the strain. However, the dashpot behaves as viscous flow, and its deformation rate depends on the stress acting on it. At the instant of unloading, the dashpot still bears a certain viscous stress; for the strain to retract, a reverse viscous resistance would have to be applied. Under pulse unloading, there is insufficient reverse driving stress to make the dashpot flow back immediately. Because the dashpot cannot contract instantaneously and is strongly bound to the spring in parallel, the spring is also held back, so elastic rebound is suppressed. Consequently, once unloading begins, the total strain cannot immediately decrease; as the stress gradually decreases, the system continues to extend slightly along the strain axis and only later retracts slowly, forming a wide, rightward-extending unloading loop in the stress–strain curve. This is inconsistent with physical tests on filled fractures, where soft infill particles do not continue to slip during pulse unloading, and the deformation should be locked immediately; in the KVM, by contrast, the parallel constraint still allows positive viscous flow in the dashpot during unloading, incorrectly producing additional viscous deformation and a nonphysical rightward extension.

When a stress wave impinges on a filled fracture, it is first loaded to produce a deformation, and then it is unloaded to rebound part of this deformation. The negative component of the reflected waveform can indirectly represent this rebounding process. Given that the deformation of the filled fracture complies with the PEM, the elastic deformation in the loading stage can be completely rebounded in the unloading stage. Thus, the negative component of the reflected wave is comparable to the positive component, as shown in Figure 5. However, it is believed that deformation of a natural filled fracture by dynamic stressing cannot recover completely when unloaded. For the KVM, because the dashpot is locked into synchronous motion with the spring and cannot retract immediately during unloading, the predicted reflected stress wave has basically no negative component. This suggests that no rebounding deformation occurs at all. Therefore, the unloading stress predicted by the KVM decreases while the strain increases, as shown in Figure 8, which is unreasonable for characterizing the response of an actual filled fracture to dynamic stressing.

By contrast, the MVM is a spring-dashpot series configuration, in which the spring and dashpot share the same stress while their strains are independent. At the instant of unloading, the spring can independently complete elastic rebound, while the viscous strain already accumulated in the dashpot remains unchanged and no new viscous flow occurs. The total strain therefore undergoes only a small recovery of the elastic component, with the dashpot locking most of the deformation. The stress–strain curve thus shows a nearly vertical unloading path and can reproduce the trapped unloading effect of filled fractures well. Due to the trapped-unloading response, only a limited part of the deformation by dynamic stressing can be rebounded, which is similar to the actual situation compared with the PEM and KVM. Therefore, the MVM can characterize the response of a filled fracture to dynamic stressing well.

### 4.2. Hydromechanical Process

When the infill is dry, the response of the filled fracture to dynamic stressing should also be characterized by the MVM. This suggests that interactions among dry granular particles could also result in viscosity in addition to liquid viscous coupling. The experimental results in Section 3 showed that variation in the water content of the infill has very significant effects on the dynamic response of the filled fracture. As the water content was below 9.45%, the water volume was too small to bear loads and could only coat particles, which reduced the friction of the contacts. This could favor rolling and slipping among granular particles and decrease the modulus (stiffness) of the filled fracture. When the water content was greater than 9.45%, considerable pores in granular particles were filled by water, and the liquid could bear part of the loads. As the water content of the infill increased further, the compressibility significantly decreased. This hydromechanical effect can explain why the modulus (stiffness) first decreased and then increased as the water content increased. As a result, the transmission (reflection) coefficient of the stress wave through a filled fracture first decreased (increased) and then increased (decreased) as the water content of the infill increased. It is worth noting that the variation in the viscous coefficient versus the water content is similar to that of the elastic stiffness. This suggests that the viscosity of the filled fracture decreased rather than increased as the water content increased from 0% to 9.45%. Further studies should be conducted to clarify these physics. According to Equation (14) and forward-fitted properties in Table 2, stress relaxation curves related to varied water contents were acquired, as shown in Figure 12. The initial compressive stress was set to 6 MPa.

(14) $$\sigma =\sigma_{0}e^{-t/t0},$$

where *σ* denotes the relaxation stress, *σ*_0_ is the initial compressive stress, *t* stands for time, and *t*_0_ = *η*_n_/*k*_n_ is the relaxation time.

Basically, the curve in Figure 12 first becomes steeper as the water content increases from 0% to 9.45%, and then it becomes flatter when the water content increases from 14.18% to 23.63%. This suggests that increasing the water content can accelerate the trapped-unloading response on the condition that the water content is relatively low. When the water content is over a critical value, the liquid begins to bear part of the external loads, which slows down the trapped-unloading response instead of increasing the water content. The above physics can be attributed to the variation in the relaxation time *t*_0_ depending on the hydromechanical behavior of the infill. As the water content increases, the susceptibility of the viscous coefficient *η*_n_ to variations in the water content is different from that of the elastic stiffness *k*_n_. As the water content increases, *t*_0_ = *η*_n_/*k*_n_ first decreases and then increases again, which leads to the result in Figure 12.

### 4.3. Implications for the Dynamic Weakening of a Gouge-Filled Fault

Previous studies have shown that earthquakes can be triggered by dynamic stresses associated with the passage of seismic waves [26,30,31,32,33], which are related to the dynamic weakening of gouge-filled faults. From our current study, we can acquire some new implications for hydrodynamic weakening of gouge-filled faults.

In the SHPB tests, high-amplitude stress waves generated by impact loading are applied to the artificial filled fracture, and the entire loading process lasts only approximately hundreds of microseconds. Within this extremely short duration, pore water pressure rises rapidly, yet conventional pore-pressure sensors cannot achieve a sufficiently high-frequency real-time response to accurately capture the pore-pressure evolution during loading. Therefore, all analyses regarding pore water pressure in this study are mechanistic inferences based on the stress–strain response rather than direct measurements. The following pore-pressure interpretation is therefore developed within this inference framework. As shown in Figure 1, when dynamically loaded by a compressive stress wave, a gouge-filled fault was compressed, which resulted in elevated pore pressure in the granular infill. The effective stress of the gouge-filled fault can be expressed as

(15) $$\sigma^{\prime }=\sigma_{t}+\sigma_{d}-\sigma_{p},$$

where $\sigma^{\prime }$ denotes the effective stress; *σ*_t_ is tectonic stress; *σ*_d_ is the dynamic compressive stress; and *σ*_p_ is the pore pressure of the infill.

Due to the trapped-unloading response, the gouge-filled fault was trapped, which only allowed limited compressive deformation to be recovered. This suggests that the highly elevated pore pressure cannot be promptly dissipated after the passage of the stress wave. However, the dynamic compressive stress *σ*_d_ dissipated to 0. Generally, the tectonic stress *σ*_t_ is stable and basically remains invariant when influenced by the passage of the stress wave from the remote source. Therefore, the effective stress of the infill $\sigma^{\prime }$ may significantly decrease, which reduces the shear strength of the fault and ultimately favors the weakening of the gouge-filled fault. Previous studies qualitatively concluded that an increase in pore pressure related to the dynamic deformation response inside a fault gouge could reduce the effective stress and further weaken the shear strength of the fault. However, there is a lack of physical models to clarify this dynamic deformation response resulting in hydrodynamic effects on fault weakening. In this study, we suggest that an MVM can characterize the response of a gouge-filled fault to dynamic stressing. Within this inference framework, although direct pore-pressure monitoring was not possible in the SHPB tests, the trapped-unloading effect inferred from stress relaxation provides indirect implications for the hydrodynamic mechanism of dynamic fault weakening. Given that elevated pore pressure is central to this mechanism, future work should focus on developing pore-pressure measurement techniques suitable for granular materials under high-speed loading and on conducting experiments on pore-pressure evolution in gouge-filled faults under dynamic loading. Cross-validation with numerical simulations would help constrain and test the assumptions adopted in the MVM. If necessary, a pore-pressure coupling term can be incorporated into the model. If current experimental conditions are insufficient for direct measurement, indirect experiments combined with numerical inversion can provide preliminary validation.

### 4.4. Limitations of the Current Study

The main purpose of this study is to explore the trapped-unloading response of filled fractures to dynamic stressing and to further acquire implications for the dynamic weakening of gouge-filled faults. Granular filling materials tend to undergo lateral deformation under dynamic loading. Without lateral confinement, the granular medium would squeeze out from the test assembly. Therefore, the aluminum-alloy sleeve was adopted to maintain a one-dimensional dynamic compression condition. We acknowledge that the sleeve indeed exerts a notable influence on the measured stiffness and pore-pressure dissipation behavior. Nevertheless, technical challenges still remain to fully eliminate such confinement effects under the present dynamic test configuration. It should be noted that the current study ignored the effect of the initial tectonic stress state and rate effect of geological materials. Although the simple MVM can characterize experimental results on dynamic compression well, a natural gouge-filled fault is far more complicated than the simulated gouge-filled fault, especially for the shear loading case. Thus, in future work, more complete and advanced models should be proposed to characterize the seismic response of an actual gouge-filled fault under both compression and shearing conditions, comprehensively considering the initial tectonic stress state and rate effect.

## 5. Conclusions

This work consolidates key quantitative physical phenomena and viscoelastic mechanical behaviors of filled fractures under dynamic stressing. The experimental observations reveal that filled fractures undergo pronounced compression during dynamic loading and exhibit negligible rebound deformation in the unloading stage, presenting a distinctive trapped-unloading feature and prominent stiffness enhancement after loading. The dynamic deformation and stress relaxation characteristics of such fracture systems can be interpreted and approximated well by the MVM. The results further verify that changes in infill water content serve as a core controlling factor that comprehensively regulates the stiffness, viscous properties, and stress relaxation responses of filled fractures, which further governs stress wave propagation and the overall dynamic deformation behaviors of fracture media. It is inferred that higher water content may substantially impede the dissipation of excess pore water pressure, lower the effective confining stress, and ultimately exacerbate the hydromechanical weakening of filled fractures. The quantitatively established correlation between infill hydraulic conditions and dynamic hydromechanical performances, as well as the validated Maxwell-based viscoelastic characterization, provides innovative mechanistic understanding of the dynamic response of filled fractures and offers fresh experimental insights into the dynamic weakening mechanism of natural gouge-filled faults under dynamic stressing.

## Figures and Tables

**Figure 1:**
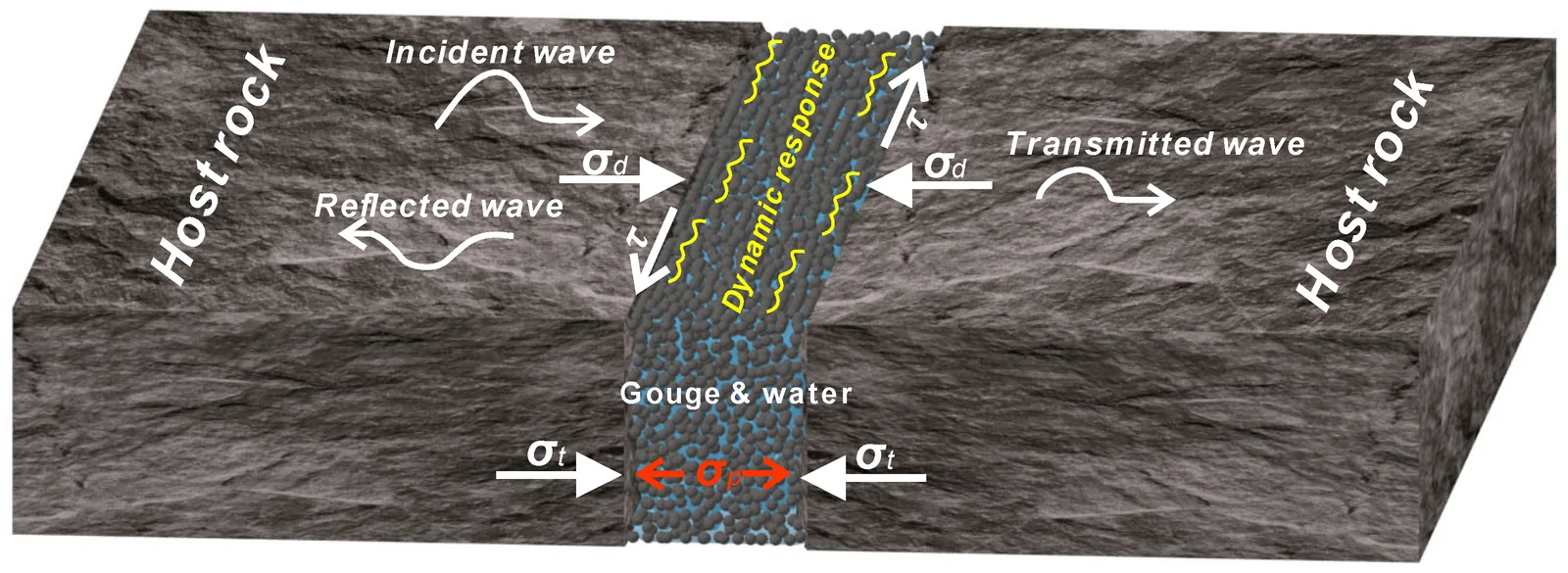
Schematic view of the response of a filled fracture (joint or fault) to dynamic stressing. When impinging on a filled fracture, only one part of the stress wave can be transmitted, while one part is reflected, resulting in energy attenuation and the slowing down of the wave velocity. Concurrently, transient loading leads to a dynamic deformation response of the filled fracture, which can elevate the pore pressure of granular materials. Elevated pore pressure counteracts parts of the compressive stress and reduces the effective stress of the filled fracture. As a result, the filled fracture has a weakened shear strength and loses stability, ultimately triggering the instability of the engineering rock mass or even earthquakes.

**Figure 2:**
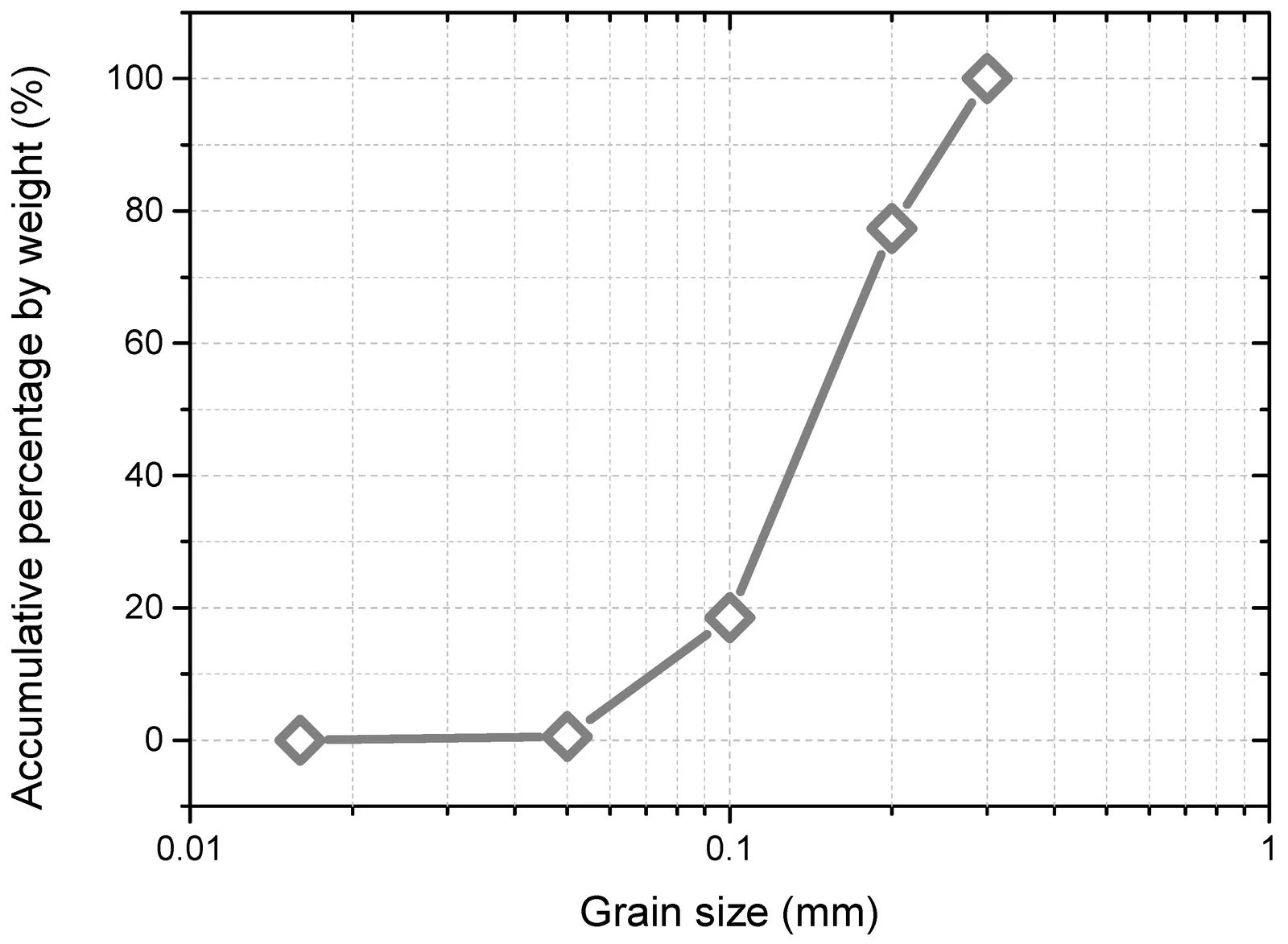
Grain size distribution of the granular material.

**Figure 3:**
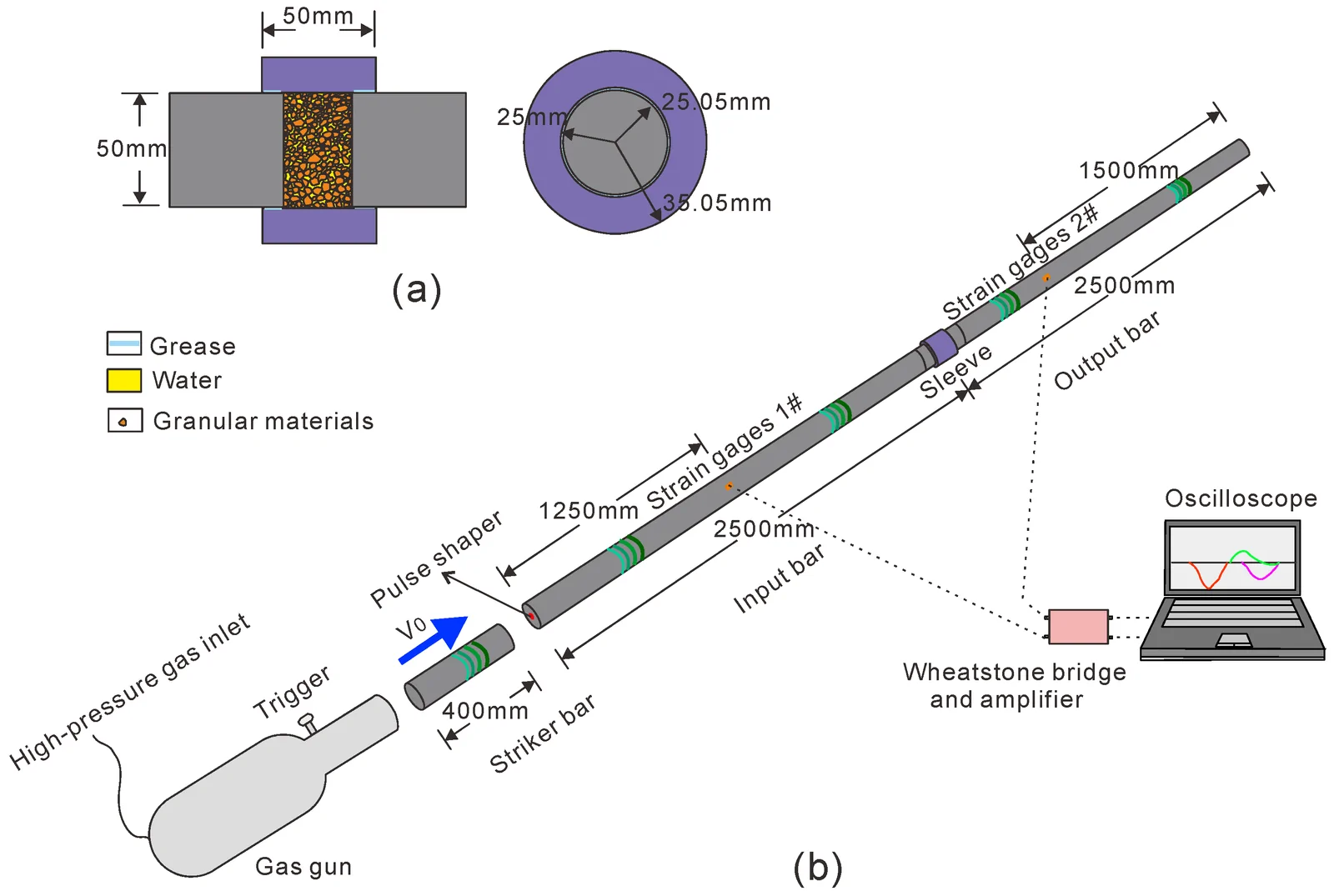
Schematic views of (**a**) the artificial filled fracture and (**b**) the dynamic stressing setup. The striker bar was pushed out with a certain velocity by the sudden release of pressurized air from a gas gun. Then, it impacted the input bar end to induce a stress pulse. The rubber pulse shaper was mounted on the input bar end to excite approximately half-sinusoidal waves. Dynamic stress was transmitted to the granular infill via the input bar and then into the output bar. The incident, reflected and transmitted stress waves were recorded by two pairs of strain gauges mounted on the incident and output bars. Signals from each pair of strain gauges were conditioned with a Wheatstone bridge to ensure the precision of the testing data. Data were acquired at 20 MHz with 12-bit digital resolution.

**Figure 4:**
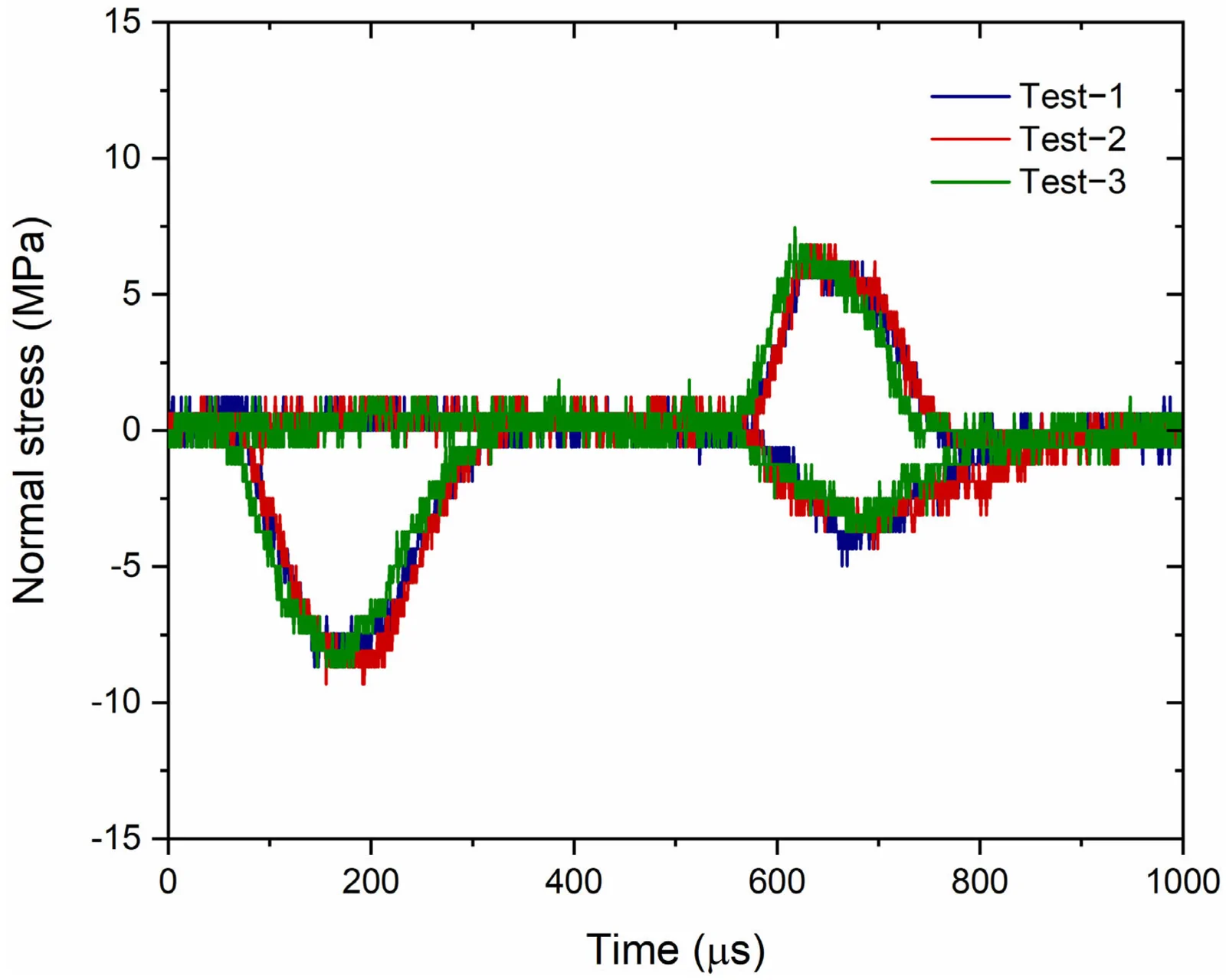
Three repeated tests: induced incident stress waveforms together with the corresponding reflected and transmitted stress waveforms upon impact on filled fractures at an infill water content of 18.90% and SHPB gas driving pressure of 0.16 MPa.

**Figure 5:**
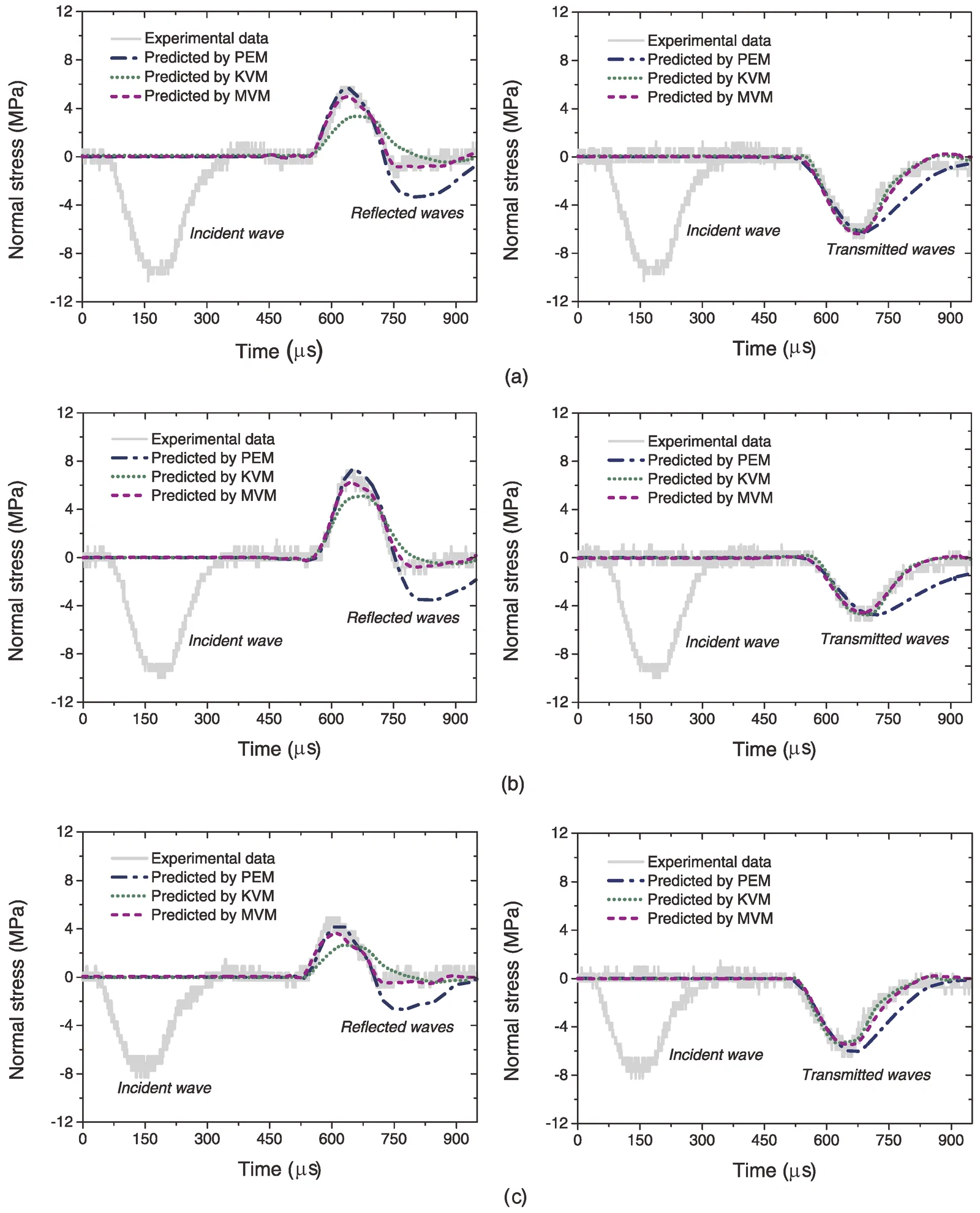
Experimental and predicted stress waveforms considering different water contents of the infill: (**a**) 0% (dry); (**b**) 9.45%; and (**c**) 23.63% (saturated). Note that the raw experimental data were strongly affected by noise; appropriate smoothing and averaging were therefore applied before model calculations and predictions to mitigate the effects of noise.

**Figure 6:**
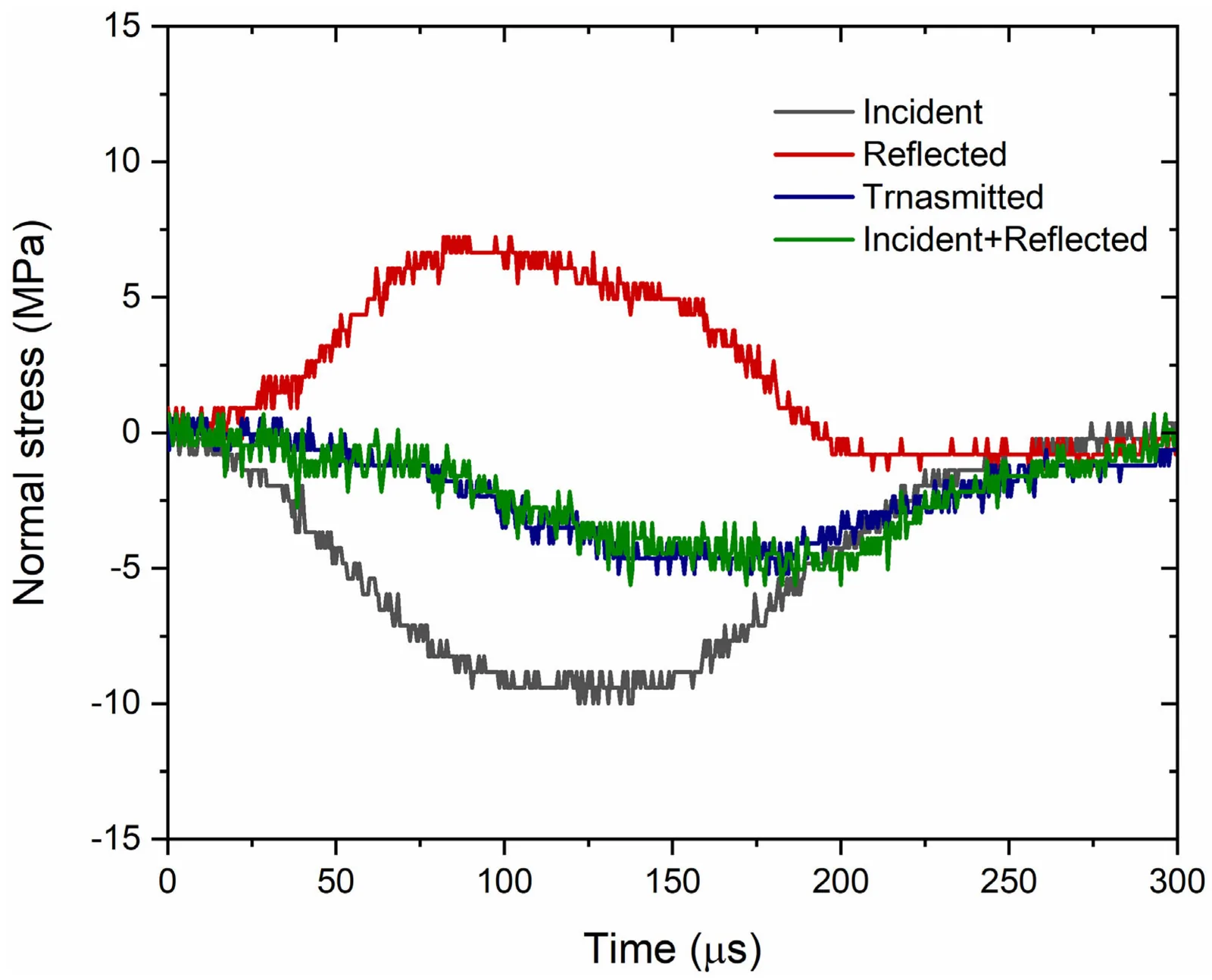
Stress balance check at the front and rear interfaces of the filled fracture corresponding to the infill water content of 9.45%.

**Figure 7:**
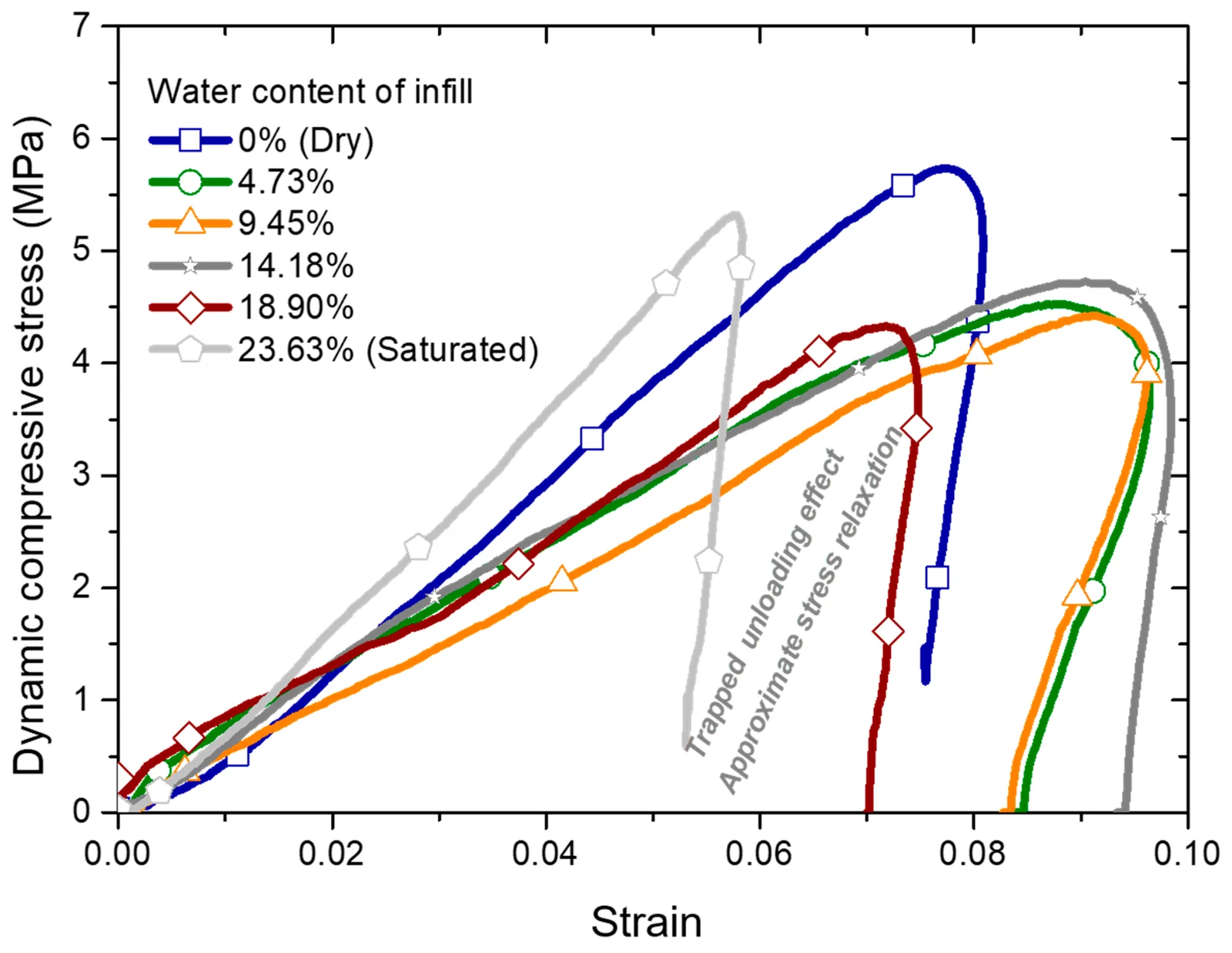
Stress–strain relations of filled fractures by dynamic stressing. Note that a filled fracture was compressed by dynamic stressing, and then it was basically trapped when unloaded, with only limited rebound deformation. Note that the raw experimental data were strongly affected by noise; appropriate smoothing and averaging were therefore applied before calculating the stress–strain relationship to mitigate the effects of noise.

**Figure 8:**
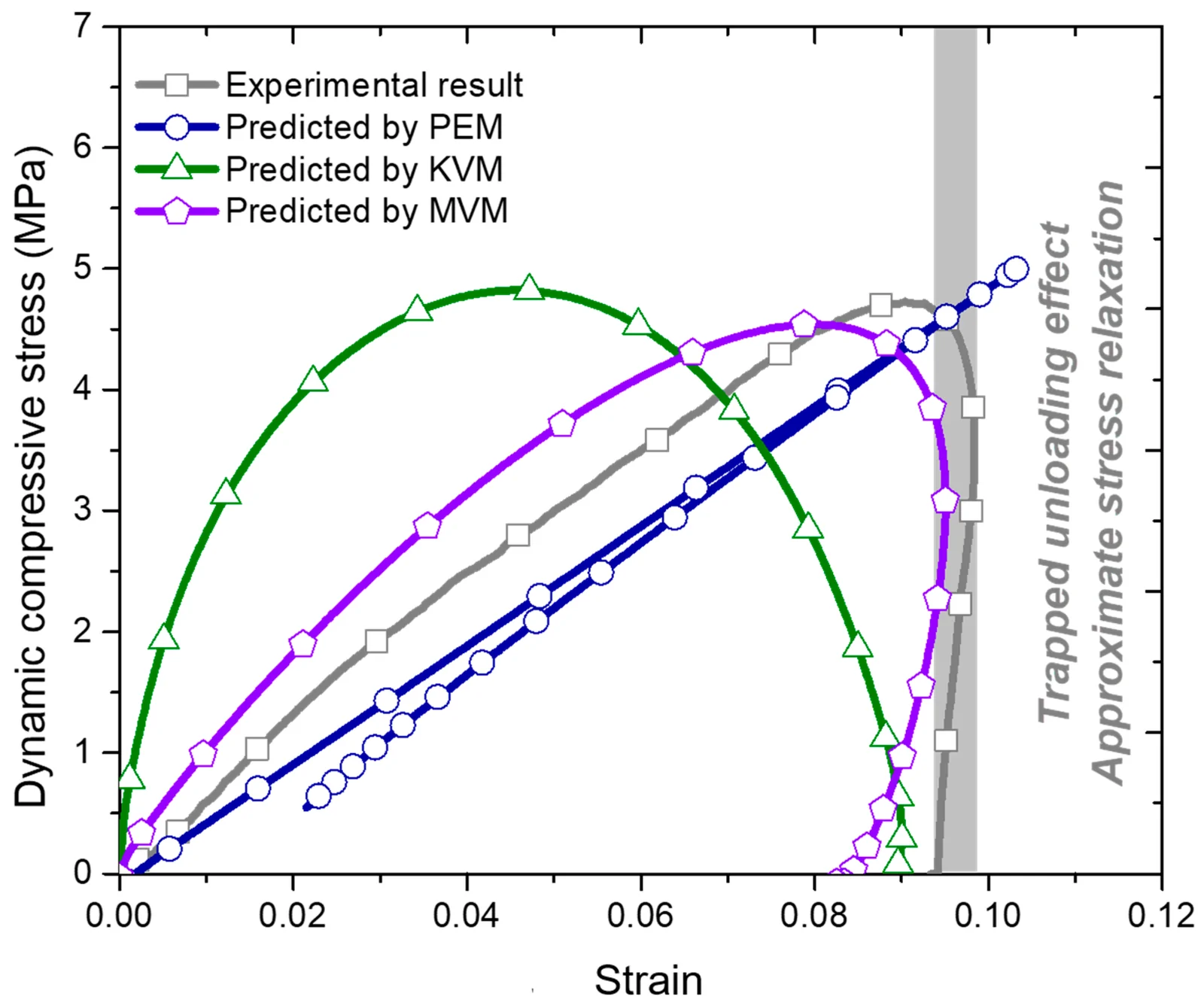
Experimental and predicted stress–strain relations when the water content of the infill is 14.18%. Note that the raw experimental data were strongly affected by noise; appropriate smoothing and averaging were therefore applied before model calculations and predictions to mitigate the effects of noise.

**Figure 9:**
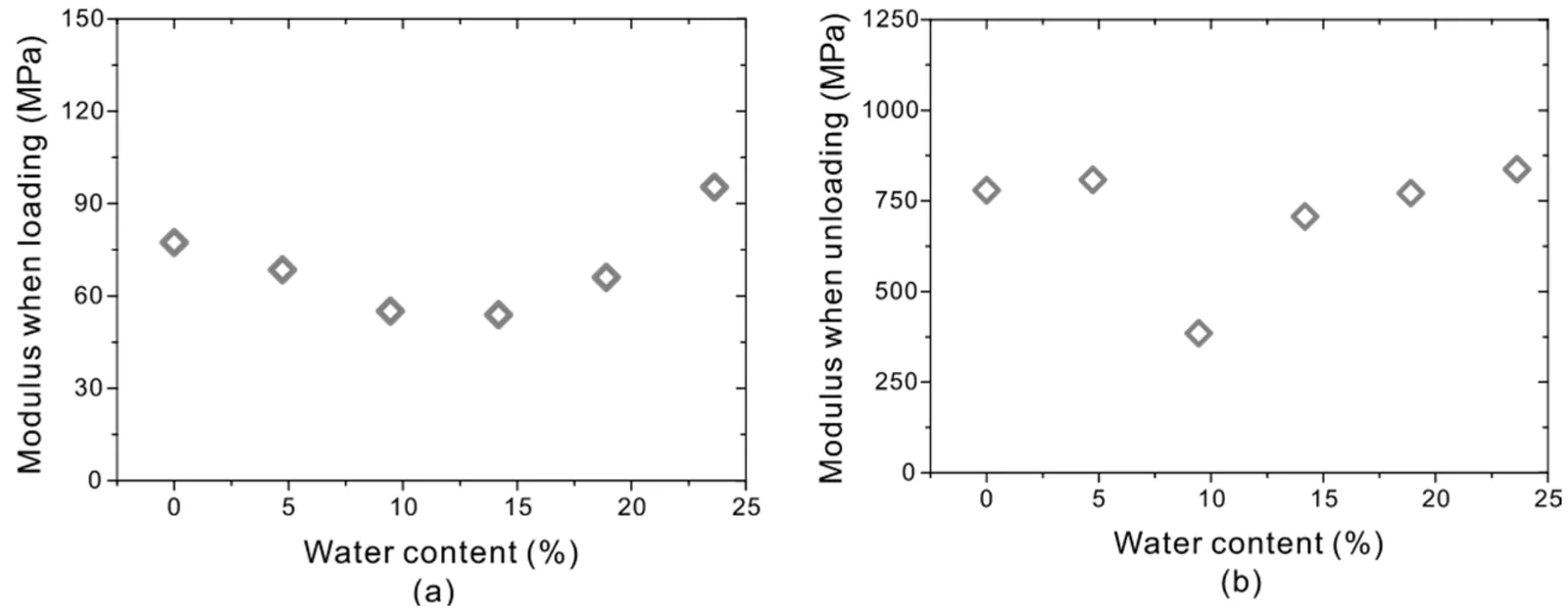
Modulus of the filled fracture considering varied water contents of the infill: (**a**) results during loading; (**b**) results during unloading.

**Figure 10:**
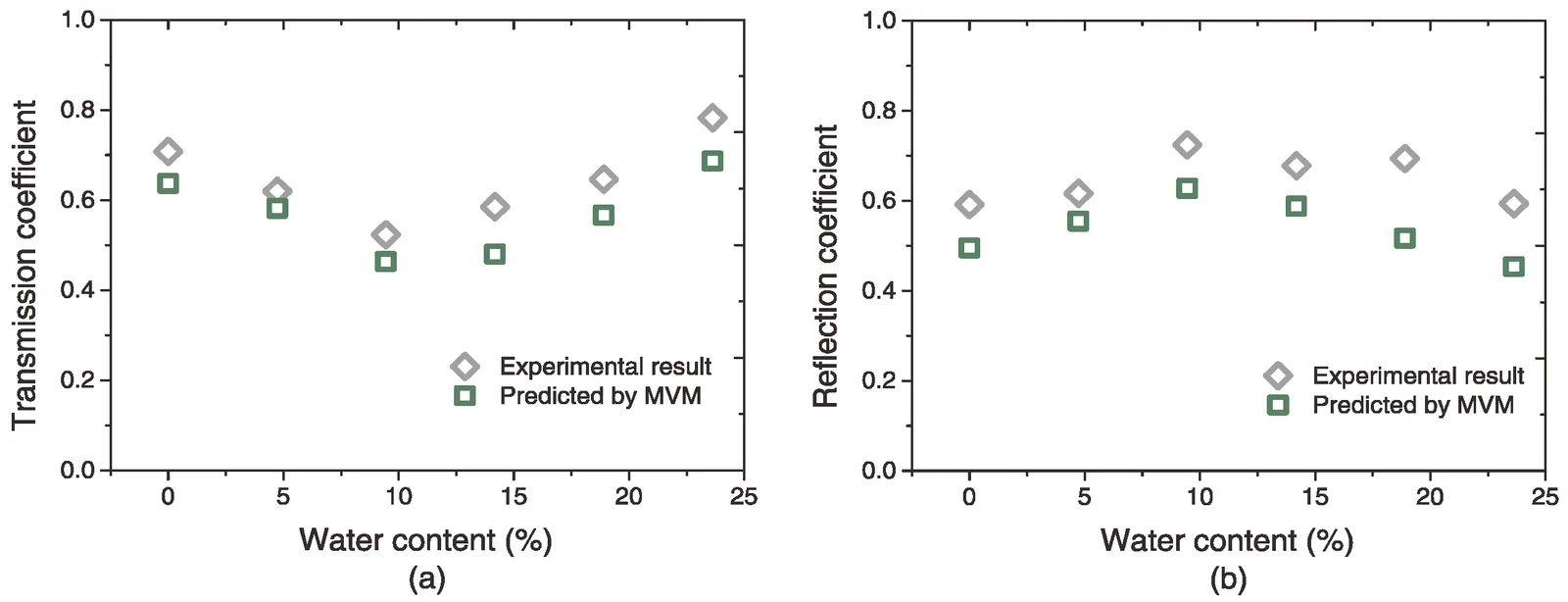
Variation in (**a**) the transmission coefficient and (**b**) the reflection coefficient of the stress wave impinging on filled fractures with varied water contents.

**Figure 11:**
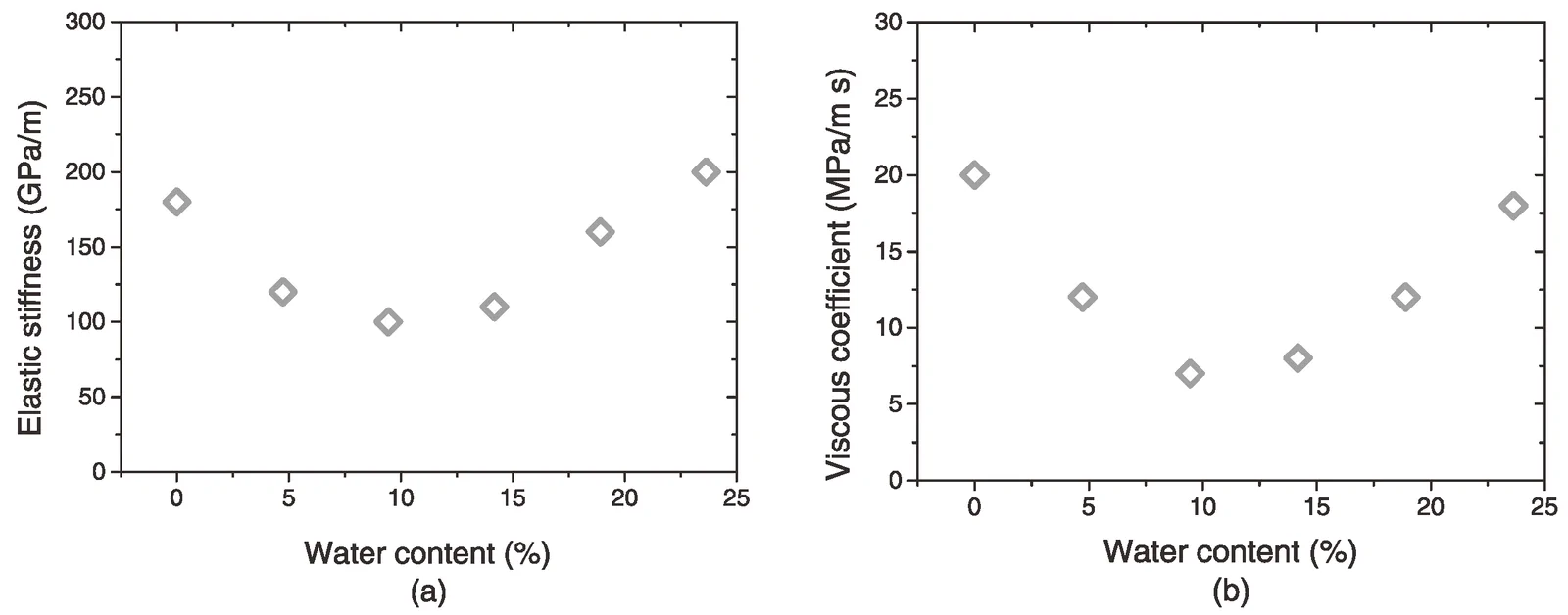
Properties related to the stress relaxation of the filled fracture forward-fitted by the MVM as the water content of the infill ranges from 0% (dry) to 23.63% (saturated): (**a**) elastic stiffness; (**b**) viscous coefficient.

**Figure 12:**
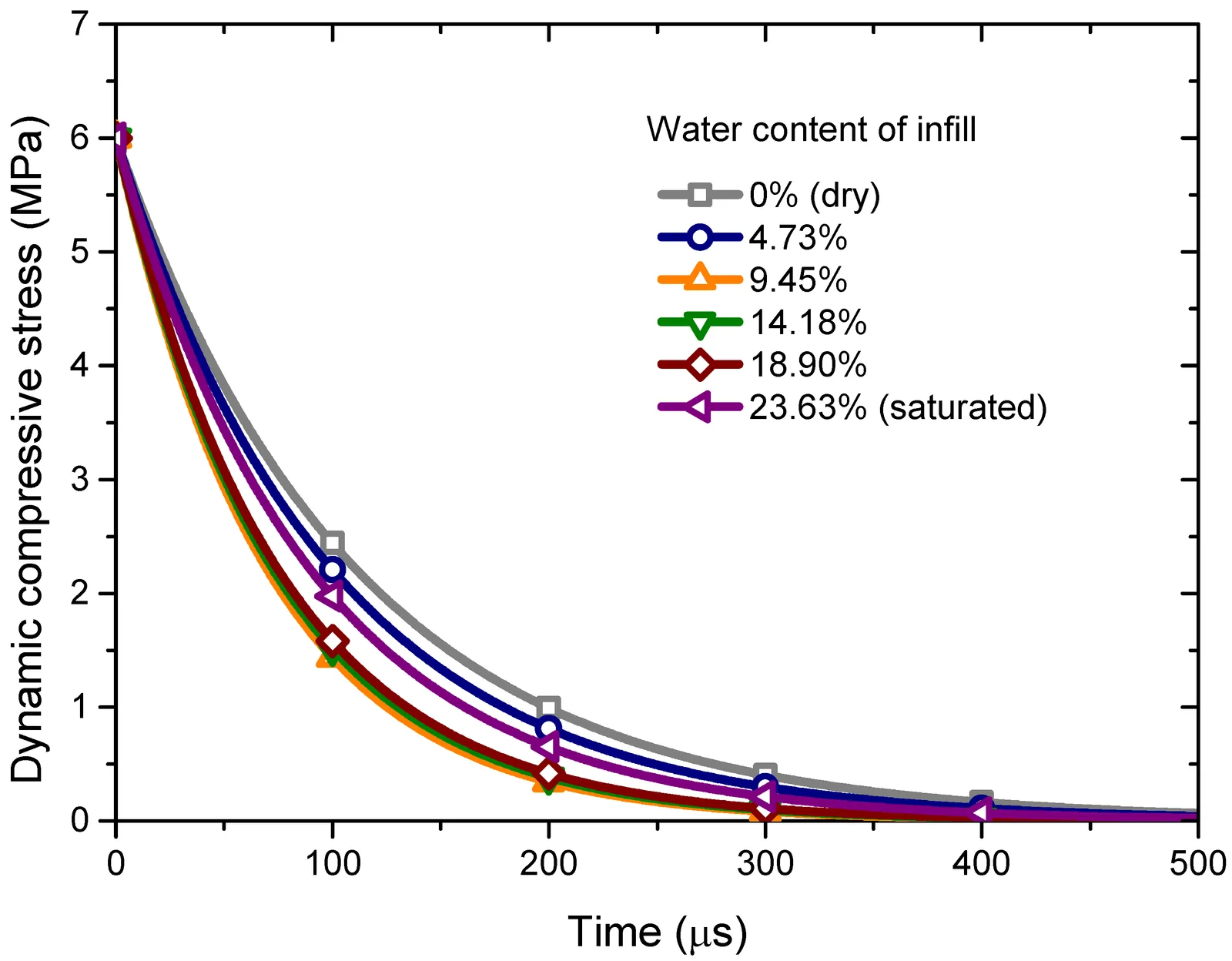
Stress relaxation curves of the filled fracture considering various water contents of the infill.

**Table 1:** Physical properties of granular infills related to this study.

Water Content (%)	Density (kg/m^3^)	Filled Thickness (mm)
0	1750	2.6
4.73	1824	2.5
9.45	1906	2.5
14.18	1988	2.5
18.90	2071	2.6
23.63	2153	2.5

**Table 2:** Properties of artificial filled fractures forward-fitted by different models.

Water Content of Infill (%)	PEM	KVM		MVM	
	*k*_n_ (GPa/m)	*k*_n_ (GPa/m)	*η*_n_ (MPa/m∙s)	*k*_n_ (GPa/m)	*η*_n_ (MPa/m∙s)
0	100	25	20	180	20
4.73	50	25	6	120	12
9.45	40	5	6	100	7
14.18	50	5	7	110	8
18.90	60	9	10	160	12
23.63	100	12	13	200	18

**Table 3:** Prediction accuracy of three constitutive models for reflected and transmitted stress waves based on R^2^ and NRMSE calculated from Figure 5.

Water Contents of the Infill	Metric	Reflected Waves			Transmitted Waves		
		PEM	KVM	MVM	PEM	KVM	MVM
0%	R^2^	0.5313	0.6710	0.9580	0.6373	0.9420	0.8955
	NRMSE	0.2091	0.1752	0.0626	0.1968	0.0785	0.1053
9.45%	R^2^	0.7648	0.8478	0.9672	0.4297	0.9594	0.9635
	NRMSE	0.1614	0.1294	0.0601	0.2477	0.0661	0.0626
23.63%	R^2^	0.4700	0.5108	0.9257	0.6474	0.9616	0.9090
	NRMSE	0.2310	0.2219	0.0850	0.1898	0.0628	0.0964

Note: R^2^ is the coefficient of determination, and values closer to 1 indicate better performance; NRMSE is the normalized root mean square error, and smaller values indicate better performance.

**Figure A1:**
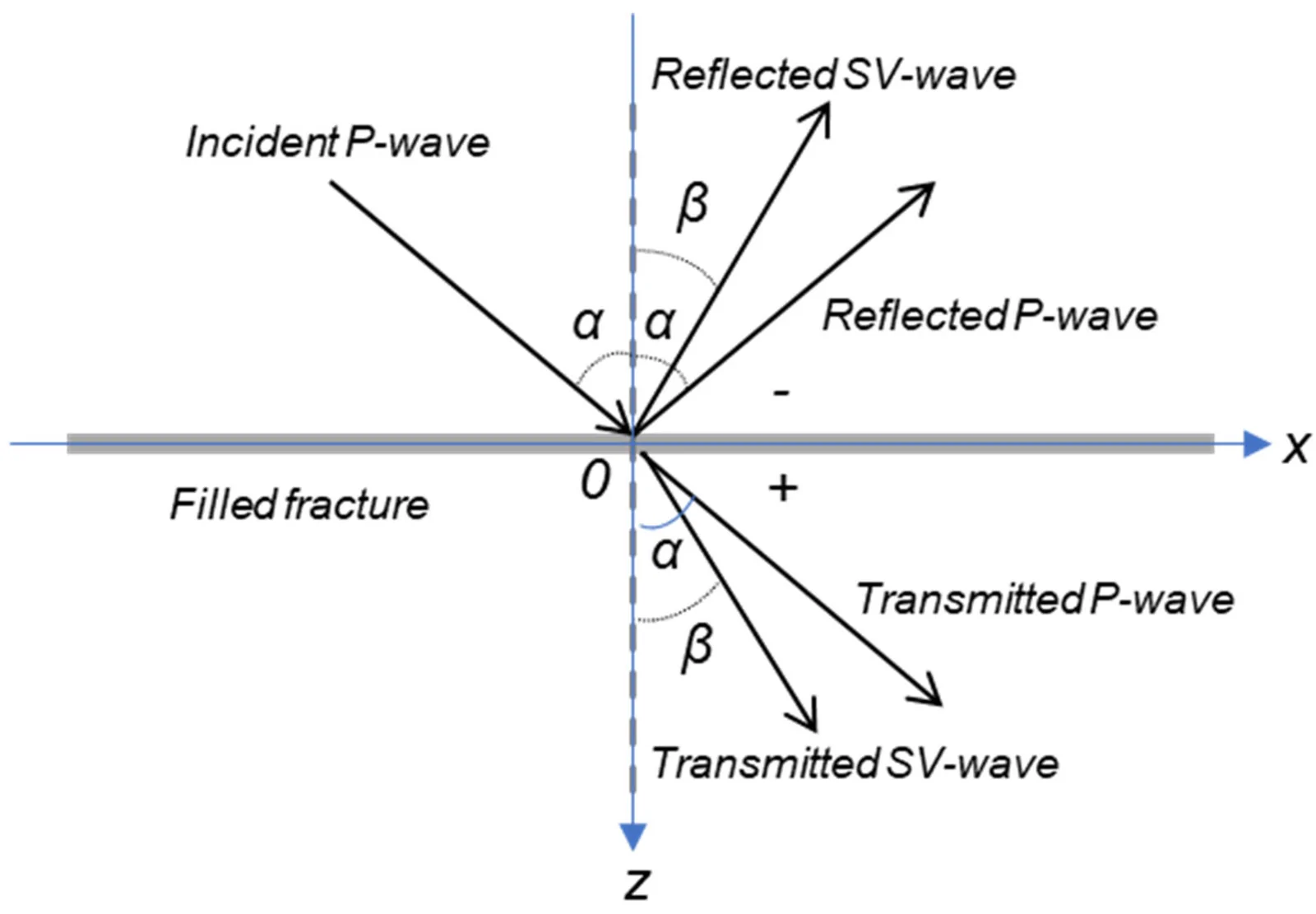
Reflection and transmission of a P-wave impinging on a filled fracture. An x–z coordinate system is adopted, where the filled fracture coincides with the *x*-axis. The “−”an “+” and denote the rock interfaces on the two sides of the filled fracture. α and β represent the reflection angles of the P-wave and SV-wave, respectively.

## Data Availability

Data used in this paper are available from Huang [53].

## Abbreviations

The following abbreviations are used in this manuscript:

KVM	Kelvin viscoelastic model
MVM	Maxwell viscoelastic model
PEM	purely elastic model
PMM	propagator matrix method
SHPB	split Hopkinson pressure bar

## Appendix A

Huang et al. [50] established an analytical model for stress waves across unfilled Kelvin viscoelastic fractures using the propagator matrix method [54]. Adopting a similar methodology, this study derives corresponding analytical models for stress wave propagation through filled fractures with pure elastic, Kelvin viscoelastic, and Maxwell viscoelastic properties. As illustrated in Figure A1, consider the reflection and transmission behaviors of P-waves incident on a single filled fracture. The detailed derivation procedures are presented as follows:

Assuming the rock behaves as a purely linear-elastic medium, with density *ρ_r_*, P-wave velocity *V_p_*, and S-wave velocity *V_s_*, the 2D wave equations are given by

(A1) $$\rho_{r}\frac{\partial^{2}u_{x}}{\partial t^{2}}=\frac{\partial \sigma_{xx}}{\partial x}+\frac{\partial \sigma_{xz}}{\partial z}$$

(A2) $$\rho_{r}\frac{\partial^{2}u_{z}}{\partial t^{2}}=\frac{\partial \sigma_{xz}}{\partial x}+\frac{\partial \sigma_{zz}}{\partial z}$$

(A3) $$\sigma_{zz}=\lambda \frac{\partial u_{x}}{\partial x}+(\lambda +2\mu )\frac{\partial u_{z}}{\partial z}$$

(A4) $$\sigma_{xz}=\mu (\frac{\partial u_{z}}{\partial x}+\frac{\partial u_{x}}{\partial z})$$

where *u_i_* is the particle displacement component; *σ_ij_* denotes the stress component; *λ* and *μ* are the Lamé constants of the rock material, with *λ* = *ρ*(*V_p_*^2^ − 2*V_s_*^2^) and *μ* = *ρV_s_*^2^.

By applying the Fourier transform to convert Equations (A1)–(A4) into the frequency domain, we obtain

(A5) $$-\rho_{r}\omega^{2}{\tilde{u}}_{x}=(\frac{\partial {\tilde{\sigma }}_{xx}}{\partial x}+\frac{\partial {\tilde{\sigma }}_{xz}}{\partial z})$$

(A6) $$-\rho_{r}\omega^{2}{\tilde{u}}_{z}=(\frac{\partial {\tilde{\sigma }}_{xz}}{\partial x}+\frac{\partial {{\tilde{\sigma }}_{z}}_{z}}{\partial z})$$

(A7) $${\tilde{\sigma }}_{zz}=\lambda \frac{\partial {\tilde{u}}_{x}}{\partial x}+(\lambda +2\mu )\frac{\partial {\tilde{u}}_{z}}{\partial z}$$

(A8) $${\tilde{\sigma }}_{xz}=\mu (\frac{\partial {\tilde{u}}_{x}}{\partial z}+\frac{\partial {\tilde{u}}_{z}}{\partial x})$$

where *ω* is the angular frequency; variables with a tilde symbol ~ above them denote their Fourier-transformed counterparts.

Based on the Helmholtz decomposition theory [55,56], the two-dimensional dynamic equations for a purely elastic medium can be represented by two displacement potential functions, namely *ϕ* (P-wave displacement potential) and *ψ*_2_ (the second component of the S-wave displacement potential). Zhao et al. [55] identified four wave modes within the rock layer and formulated the time-domain expressions for the layer-related potentials *ϕ* and *ψ*_2_ as

(A9) $$\phi (x,z,t)=U_{p}e^{j(\omega t-k_{x}x+k_{zp}z)}+D_{p}e^{j(\omega t-k_{x}x-k_{zp}z)}$$

(A10) $$\psi_{2}(x,z,t)=U_{s}e^{j(\omega t-k_{x}x+k_{zs}z)}+D_{s}e^{j(\omega t-k_{x}x-k_{zs}z)}$$

where $j=\sqrt{-1}$ is the imaginary unit; *k_x_* and *k_zp_* are the P-wave numbers in the x- and z-directions, and *k_zs_* denotes the S-wave number in the z-direction. For P-wave incidence, the wave numbers *k_x_*, *k_zp_* and *k_zs_* are given by *k_x_* = *k_p_sinα*, $k_{zp}=\sqrt{{\left(\omega /V_{p}\right)}^{2}-{k_{x}}^{2}}$, $k_{zs}=\sqrt{{\left(\omega /V_{s}\right)}^{2}-{k_{x}}^{2}}$, where *k_p_* = *ω*/*V_p_* is the wavenumber of the downgoing P-wave and *α* is the incident angle of the stress wave. *U_p_*, *D_p_*, *U_s_* and *D_s_* represent the displacement-potential amplitudes of the up-going P-wave, downgoing P-wave, up-going S-wave, and downgoing S-wave, respectively.

The components of particle displacement can be expressed as

(A11) $$u_{x}=\frac{\partial \phi }{\partial x}-\frac{\partial \psi_{2}}{\partial z}$$

(A12) $$u_{z}=\frac{\partial \phi }{\partial z}+\frac{\partial \psi_{2}}{\partial x}$$

By applying the Fourier transform to convert Equations (A9)–(A12) into the frequency domain, we obtain

(A13) $$\tilde{\phi }(x,z,\omega )=U_{p}e^{j(-k_{x}x+k_{zp}z)}+D_{p}e^{j(-k_{x}x-k_{zp}z)}$$

(A14) $${\tilde{\psi }}_{2}(x,z,\omega )=U_{s}e^{j(-k_{x}x+k_{zs}z)}+D_{s}e^{j(-k_{x}x-k_{zs}z)}$$

(A15) $${\tilde{u}}_{x}=\frac{\partial \tilde{\phi }}{\partial x}-\frac{\partial {\tilde{\psi }}_{2}}{\partial z}$$

(A16) $${\tilde{u}}_{z}=\frac{\partial \tilde{\phi }}{\partial z}+\frac{\partial {\tilde{\psi }}_{2}}{\partial x}$$

Substituting Equations (A13) and (A14) into Equations (A15) and (A16), the frequency-domain particle displacement components at arbitrary positions within the rock layer are obtained.

(A17) $${\tilde{u}}_{x}=[(-jk_{x}e^{jk_{zp}z})U_{p}+(-jk_{x}e^{-jk_{zp}z})D_{p}+(-jk_{zs}e^{jk_{zs}z})U_{s}+(jk_{zs}e^{-jk_{zs}z})D_{s}]e^{-jk_{x}x}$$

(A18) $${\tilde{u}}_{z}=[(jk_{zp}e^{jk_{zp}z})U_{p}+(-jk_{zp}e^{-jk_{zp}z})D_{p}+(-jk_{x}e^{jk_{zs}z})U_{s}+(-jk_{x}e^{-jk_{zs}z})D_{s}]e^{-jk_{x}x}$$

Based on Equations (A7), (A8), (A17) and (A18), the following expressions are obtained

(A19) $${\tilde{\sigma }}_{xz}=\left[\begin{array}{c} 2\mu k_{x}k_{zp}e^{jk_{zp}z}U_{p}+2\mu (-k_{x}k_{zp})e^{-jk_{zp}z}D_{p}\\ -\;\mu (k_{x}^{2}-k_{zs}^{2})e^{jk_{zs}z}U_{s}-\mu (k_{x}^{2}-k_{zs}^{2})e^{-jk_{zs}z}D_{s} \end{array}\right]e^{-jk_{x}x}$$

(A20) $${\tilde{\sigma }}_{zz}=\left[\begin{array}{c} (-\frac{\lambda \omega^{2}}{V_{p}^{2}}-2\mu k_{zp}^{2})e^{jk_{zp}z}U_{p}+(-\frac{\lambda \omega^{2}}{V_{p}^{2}}-2\mu k_{zp}^{2})e^{-jk_{zp}z}D_{p}\\ +\;2\mu k_{x}k_{zs}e^{jk_{zs}z}U_{s}+2\mu (-k_{x}k_{zs})e^{-jk_{zs}z}D_{s} \end{array}\right]e^{-jk_{x}x}$$

Equations (A17)–(A20) may be expressed in matrix form

(A21) $$\left[\begin{array}{c} {\tilde{u}}_{x}\\ {\tilde{u}}_{z}\\ {\tilde{\sigma }}_{zz}\\ {\tilde{\sigma }}_{xz} \end{array}\right]=\left[P\right]\left[H\right]\left[\begin{array}{c} U_{p}\\ D_{p}\\ U_{s}\\ D_{s} \end{array}\right]e^{-jk_{x}x}$$

where

$$\left[P\right]=\left[\begin{array}{cccc} -jk_{x} & -jk_{x} & -jk_{zs} & jk_{zs}\\ jk_{zp} & -jk_{zp} & -jk_{x} & -jk_{x}\\ \left(-\frac{\lambda \omega^{2}}{V_{p}^{2}}-2\mu k_{zp}^{2}\right) & \left(-\frac{\lambda \omega^{2}}{V_{p}^{2}}-2\mu k_{zp}^{2}\right) & 2\mu k_{x}k_{zs} & -2\mu k_{x}k_{zs}\\ 2\mu k_{x}k_{zp} & -2\mu k_{x}k_{zp} & -\mu \left(k_{x}^{2}-k_{zs}^{2}\right) & -\mu (k_{x}^{2}-k_{zs}^{2}) \end{array}\right],$$

$$\left[H\right]=\left[\begin{array}{cccc} e^{jk_{zp}z} & 0 & 0 & 0\\ 0 & e^{-jk_{zp}z} & 0 & 0\\ 0 & 0 & e^{jk_{zs}z} & 0\\ 0 & 0 & 0 & e^{-jk_{zs}z} \end{array}\right].$$

Equation (A21) shows that the displacement components and stress components of any point in the rock layer can be expressed using the amplitudes of the potential functions.

The stress and displacement components at the front and rear interfaces of the filled fracture can be expressed as follows:

(A22) $${\left[\begin{array}{c} {\tilde{u}}_{x}\\ {\tilde{u}}_{z}\\ {\tilde{\sigma }}_{zz}\\ {\tilde{\sigma }}_{xz} \end{array}\right]}^{+}=\left[K\right]{\left[\begin{array}{c} {\tilde{u}}_{x}\\ {\tilde{u}}_{z}\\ {\tilde{\sigma }}_{zz}\\ {\tilde{\sigma }}_{xz} \end{array}\right]}^{-}$$

From Equations (A21) and (A22), the relationship of potential amplitudes between adjacent rock layers can be expressed as follows [50]:

(A23) $${\left[\begin{array}{c} U_{p}\\ D_{p}\\ U_{s}\\ D_{s} \end{array}\right]}^{+}={\left[H\right]}^{-1}{\left[P\right]}^{-1}\left[K\right]\left[P\right]\left[H\right]{\left[\begin{array}{c} U_{p}\\ D_{p}\\ U_{s}\\ D_{s} \end{array}\right]}^{-}$$

At the filled fracture, z = 0; hence, [*H*] reduces to the identity matrix, and Equation (A23) can be simplified as

(A24) $${\left[\begin{array}{c} U_{p}\\ D_{p}\\ U_{s}\\ D_{s} \end{array}\right]}^{+}={\left[P\right]}^{-1}\left[K\right]\left[P\right]{\left[\begin{array}{c} U_{p}\\ D_{p}\\ U_{s}\\ D_{s} \end{array}\right]}^{-}$$

For the upper rock layer, if down going *P* wave is selected as the incident wave, we can have *D_s_* = 0. For the lower rock layer, due to only existing downgoing *P* wave and *S* wave, we can have *U_p_* = 0 and *U_s_* = 0.

Assuming that $\left[M\right]={\left[P\right]}^{-1}\left[K\right]\left[P\right]=\left[\begin{array}{cccc} m_{11} & m_{12} & m_{13} & m_{14}\\ m_{21} & m_{22} & m_{23} & m_{24}\\ m_{31} & m_{32} & m_{33} & m_{34}\\ m_{41} & m_{42} & m_{43} & m_{44} \end{array}\right]$, the potential amplitudes of reflected waves in upper rock layer and transmitted waves in lower rock layer can be expressed as follows [50]:

(A25) $$\left[\begin{array}{c} U_{p}^{-}\\ D_{p}^{+}\\ U_{s}^{-}\\ D_{s}^{+} \end{array}\right]={\left[\begin{array}{cccc} m_{11} & 0 & m_{13} & 0\\ m_{21} & -1 & m_{23} & 0\\ m_{31} & 0 & m_{33} & 0\\ m_{41} & 0 & m_{43} & -1 \end{array}\right]}^{-1}\left[\begin{array}{cccc} -m_{12} & 0 & 0 & 0\\ 0 & -m_{22} & 0 & 0\\ 0 & 0 & -m_{32} & 0\\ 0 & 0 & 0 & -m_{42} \end{array}\right]\left[\begin{array}{c} D_{p}^{-}\\ D_{p}^{-}\\ D_{p}^{-}\\ D_{p}^{-} \end{array}\right]$$

Note that when the potential amplitude of the incident P-wave is given as *D_p_*^−^, the potential amplitudes of the reflected P-wave, reflected SV-wave, transmitted P-wave and transmitted SV-wave can be calculated by Equation (A25). Based on the above, the corresponding stresses and displacements can be computed using Equation (A21). This study considers only normal incidence (*α* = 0, *β* = 0 in Figure A1), such that no reflected and transmitted SV-waves occur.
